# Fold change rank ordering statistics: a new method for detecting differentially expressed genes

**DOI:** 10.1186/1471-2105-15-14

**Published:** 2014-01-15

**Authors:** Doulaye Dembélé, Philippe Kastner

**Affiliations:** 1 Institut de Génétique et de Biologie Moléculaire et Cellulaire (IGBMC), INSERM U964, CNRS UMR 7104, Université de Strasbourg, 67404 Illkirch, France; 2 IGBMC Microarray and Sequencing Platform, 67404 Illkirch, France; 3 Faculté de Medécine, Université de Strasbourg, Strasbourg, France

**Keywords:** Differentially expressed genes, Fold change, Averages of ranks, Microarray

## Abstract

**Background:**

Different methods have been proposed for analyzing differentially expressed (DE) genes in microarray data. Methods based on statistical tests that incorporate expression level variability are used more commonly than those based on fold change (FC). However, FC based results are more reproducible and biologically relevant.

**Results:**

We propose a new method based on fold change rank ordering statistics (FCROS). We exploit the variation in calculated FC levels using combinatorial pairs of biological conditions in the datasets. A statistic is associated with the ranks of the FC values for each gene, and the resulting probability is used to identify the DE genes within an error level. The FCROS method is deterministic, requires a low computational runtime and also solves the problem of multiple tests which usually arises with microarray datasets.

**Conclusion:**

We compared the performance of FCROS with those of other methods using synthetic and real microarray datasets. We found that FCROS is well suited for DE gene identification from noisy datasets when compared with existing FC based methods.

## Background

To select the differentially expressed (DE) genes in a microarray dataset with two biological conditions, the Fold Change (FC) which is calculated as a ratio of averages from control and test sample values was initially used [1,2]. Levels of change or cutoffs, (e.g. 0.5 for down- and 2 for up-regulated) are used and genes under/above thresholds are selected. Then, other statistical methods were introduced. Many of these methods use three steps. First, a statistical test (e.g. Student’s t-test or similar) is performed to obtain a p-value for each gene in the study. Second, these p-values are compared to a threshold which is chosen to have an acceptable False Discovery Rate (FDR), and a list of genes is obtained. Third, a selection is done from the above list using FC level thresholds for down- and up-regulated genes [3,4]. New statistical methods more adapted to microarray data were proposed [5-9]. The significance analysis of microarrays (SAM) method [6] provides an improvement to the ordinary Student t-test, as it imposes limits on the variability of genes, to exclude genes that do not change and which are associated with very low p-values. The performances of several methods were compared in [10-13] using two classes microarray datasets.

It has been shown that the FC based selection of genes leads to more reproducible results irrespective of the technology that is used [14-16]. Kadota et al. [12] proposed a FC based method, weighted average difference (WAD), which promotes highly expressed genes. WAD uses a weight factor for the average difference (AD) for each gene. The AD is obtained using log signals while the FC is computed from non-log signals. Comparative results in [12,13] show that the WAD method is powerful for detecting DE genes in microarray data. However, like the simple FC based method, WAD does not associate an error to the list of selected genes. Hence, Farztdinov and McDyer [17] proposed a distributional fold change (DFC) test using the AD. A score is computed for each gene which is used for the ranking and selection of genes. The exact statistic of the DFC score is unknown even if it allows detection of weakly expressed genes that are lost with the WAD method. To take into account the variability in gene expression levels, many statistical methods were proposed. Some of these methods include the FC information to avoid the three step selection procedure mentioned above. McCarthy et al. [18] directly include a threshold for the gap between the averages in the Student t-test: t-test relative to a threshold (TREAT). The TREAT method is based on the linear model in [9]. In [19], the FC is combined with the hypothesis testing for assessing prediction error in the selection of the DE genes. More recently, Xiao et al. [20] combined the FC with a two samples statistical test p-value to obtain a score they called *π*-value. In all methods that can calculate a probability for each gene independently of the others, the problem of multiple tests arises. To avoid this problem, Qi et al. [21] used a mixture model. This model has four components corresponding to the expression status (yes or no) for the two biological conditions for each gene in the dataset. After model parameters estimation via an Expectation-Maximization type algorithm, the probabilities associated with genes are sorted, and a threshold is used to determine the list of genes given an error level. The method we propose here also avoids the problem of multiple tests and has a lower computational load.

Breitling et al. [22] devised a statistical method based only on the FC information. In their method, the FCs obtained in multiple pairs of control/test samples are ranked in decreasing order, and the product of the ranks (RP) for each gene is calculated. Combined probabilities *p*^′^ are calculated by multiplying the RP values by a scalar factor which is determined using a permutation analysis to obtain an approximation of the expected RP values, see [22] for more details. A percentage of false-positive (PFP) is associated to each gene, and an acceptable PFP value is chosen to define the list of the DE genes. To select down-regulated genes the sorting is done in increasing order and all subsequent steps are modified accordingly. The quality of the selection of the DE genes using the rank products method will depend mainly on the quality of the approximation of the expectation of the RP values using a permutation analysis. More recently, an exact statistic was proposed for the RP method [23]. However, for data with a large number of samples, the computational load is very heavy and this method is thus not recommended. Here, we propose another method based only on FC ranks. This method is very fast in comparison to the RP method, even for large numbers of samples in the dataset. In our method it is not necessary to search for up- and down-regulated genes in two separate steps. The statistic we obtain for each gene gives direct information on its status: down-regulated, up-regulated or not changed. This method also solves the problem of multiple tests which is usually encountered for microarray datasets. We exploit variations in the FC using several pairs of control/test samples. A statistics is associated to each gene, which results from the variation of the rank and the level of its FC.

## Methods

### Preliminaries

We consider a two conditions microarray experiment where *n* probes (genes) are used with *m*_1_ control and *m*_2_ test samples. The number *n* of probes is generally greater than 10,000 except for few species like yeast. Values for *m*_1_ and *m*_2_ are however small, most often lower than 100. We note ${\text{x}}_{i}=({\text{x}}_{i}^{c},{\text{x}}_{i}^{t})=(x_{i1}^{c}x_{i2}^{c}\ldots x_{im_{1}}^{c}x_{i1}^{t}x_{i2}^{t}\ldots x_{im_{2}}^{t}$) the values for the gene *i* (*i* = 1,2,…,*n*) for the control samples ( $x_{\text{ij}}^{c},j=1,2,\ldots ,m_{1}$) and the test samples ( $x_{\text{ij}}^{t},j=1,2,\ldots ,m_{2}$), respectively. For a single color microarray, values ( $x_{\text{ij}}^{c},x_{\text{ij}}^{t}$) are *log*_2_ levels, while they are *log*_2_ ratios for a two-color microarray. Here are examples of *log*_2_ transformed data for two genes (*MACF1* and *TREM2*) taken from an experiment using Agilent microarrays (SurePrint, design 028004_D_F_20101102), with one color hybridization. Data for *MACF1* are: ${\text{x}}_{.}^{c}$ = (11.1435, 11.2860, 11.2249, 11.1258, 11.0325, 11.1108, 11.3377, 11.1821, 11.0675, 11.2381), ${\text{x}}_{.}^{t}$ = (11.0375, 11.0792, 10.9673, 11.0367, 11.1054, 10.9261, 11.0433, 10.9484, 10.9412, 10.8385); data for *TREM2* are: ${\text{x}}_{.}^{c}$ = (6.2856, 6.4891, 5.7799, 6.1081, 6.3129, 6.3208, 6.4826, 6.2005, 5.8922, 6.2148), ${\text{x}}_{.}^{t}$ = (11.6792, 8.1128, 6.6253, 6.8334, 7.6417, 7.5133, 5.9633, 7.4631, 6.5666, 7.6020). There are *m*_1_ = 10 control and *m*_2_ = 10 test samples. The FC and the Student t-test p-value for *MACF1* and *TREM2* are (0.8806, 0.000248) and (6.2570, 0.01259), respectively. These results lead to the following two observations: a) a small Student t-test p-value is not necessary associated to a high FC, b) a high Student t-test p-value can be associated to a high FC. Indeed, the Student t-test statistic is calculated as $t=\frac{{\bar{\text{x}}}^{t}-{\bar{\text{x}}}^{c}}{s_{p}}$, where ${\bar{\text{x}}}^{t}$ and ${\bar{\text{x}}}^{c}$ are average levels of the control and test samples respectively, $s_{p}^{2}$ is the combined variance from those of the control and test samples: $s_{p}^{2}=\frac{(m_{1}-1)s_{1}^{2}+(m_{2}-1)s_{2}^{2}}{m_{1}+m_{2}-2}$, ( $s_{1}^{2}$ and $s_{2}^{2}$ are variances of **x**^*t*^ and **x**^*c*^). For the same average difference ( ${\bar{\text{x}}}^{t}-{\bar{\text{x}}}^{c}$), a small $s_{p}^{2}$ can lead to high *t* (small p-value), on the other hand, a large $x_{p}^{2}$ can lead to a small t (high p-value). Hence, a small (high) average difference can have a small (high) Student t-test p-value. The variances of data for genes MACF1 and TREM2 given above are 0.008 and 1.26, leading to t-statitics equal to 4.549 and 2.711 respectively. These observations are highlighted by Xiao et al. [20] and correspond to the SFSV (small fold change, small variance) and the LFLV (large fold change, large variance), respectively. For the proposed method, the probability of the statistic obtained is close to zero (one) for down-(up)regulated genes. Using the method described below, the probabilities associated to the statistics obtained for *MACF1* and *TREM2* are 0.12105 and 0.9964, respectively. These values mean that MACF1 does not change and that TREM2 is up-regulated.

### Description of the method

Being given expression values for *n* genes in *m*_1_ control and *m*_2_ test samples, we perform *k* ≤ *m*_1_*m*_2_ pairwise comparisons and compute FCs for each gene (test/control). In each comparison, the *n* FCs obtained are sorted in increasing order and their corresponding ranks are associated to genes. Hence, for gene *i*, we get a vector **r**_*i*_ = (*r*_*i*1_*r*_*i*2_ … *r*_*ij*_ …,*r*_*ik*_) where *r*_*ij*_ corresponds to the rank of the FC for gene *i* in the *j* comparison (*j* = 1,…,*k*). The ranks are integers that belong to the set { 1,2,…,*n*}. To deal with ties, the rank values are adjusted in such a way that their sum reaches the same total as that reached if there is no tie. By construction, knowledge of one component of the vector **r**_*i*_ does not allow to predict the another ones. This leads to an independence of the ranks associated to pairwise comparisons. Hence, the components of the vector **r**_*i*_ can be considered as samples of the true unknown rank associated to gene *i*. Ideally, the same rank should be assigned to each gene in the *k* comparisons. The probability of this event is $\frac{1}{n^{k+1}}$ and is unlikely to happen. Hence, the averages of ranks (a.o.r) ${\bar{r}}_{i}$, *i* = 1,2,…,*n*, will vary between a minimum $a=\min_{i}\{{\bar{r}}_{i}\}$ and a maximum $b=\max_{i}\{{\bar{r}}_{i}\}$. ${\bar{r}}_{i}$ is an average of components in **r**_*i*_. We can order all the a.o.r ${\bar{r}}_{i}$ from the minimum to the maximum and write: $\bar{r}=[a,(a+\delta_{1}),(a+\delta_{1}+\delta_{2}),\ldots ,(a+\delta_{1}+\ldots +\delta_{n-2}),b]$ where scalars *δ*_*i*_ (*i* = 1,…,*n* - 1) are the differences between consecutive ordered a.o.r, and $\bar{r}$ is a vector with all ${\bar{r}}_{i}$. Without loss of generality, let us assume that the differences *δ*_*i*_ have the same value which is approximated by their mean: $\delta =\frac{b-a}{n-1}$. Hence, the ordered a.o.r ${\bar{r}}_{i}$, *i* = 1,2,…,*n*, can then be writen as: $\bar{r}=[a,(a+\delta ),(a+2\delta ),(a+3\delta ),\ldots ,(a+(n-1)\delta )]$. Our method is based on the behavior of the ordered a.o.r $\bar{r}$ and we have the following theorem.

#### 

**Theorem 1.** *When the number k of the pairwise comparisons grows, the ordered averages of ranks (a.o.r)*$\bar{r}$*have a normal distribution. The mean of this distribution is*$\frac{a+b}{2}$*and its variance is*$\frac{n^{2}-1}{12}\delta^{2}$, *where a and b are the minimum and the maximum of the observed a.o.r*$\bar{r}$, *respectively. δ is an average difference between consecutive ordered a.o.r.*$\bar{r}$.

#### 

**Proof.** We note ${\bar{r}}_{i}=\frac{1}{k}\sum_{j=1}^{k}r_{\text{ij}}$ the average of the components in **r**_*i*_. Let us note the expectation and the variance of the ranks in vector **r**_*i*_ by *E*{**r**_*i*_} = *R*_*i*_ and $\text{Var}\{{\text{r}}_{i}\}=\sigma_{R_{i}}^{2}$. Using the central limit theorem ( [24], page 259) it follows that the quantity $\frac{\sqrt{k}}{\sigma_{R_{i}}}({\bar{r}}_{i}-R_{i})$ converges to a normal distributed variable having a mean of zero and a variance of one when *k* is high. Hence, we obtain *n* normal distributed variables *R*_*i*_.

From the selection theorem ( [24], page 267), the sequence of the *n* normal variables has a normal distribution. Expectation and variance of variable *R*_*i*_ are given by

(1) $$\begin{array}{l} \;E\{R_{i}\}=\frac{1}{n}\overset{n}{\underset{i=1}{\sum }}R_{i} \end{array}$$

(2) $$\begin{array}{l} \text{Var}\{R_{i}\}=E\{R_{i}^{2}\}-{\left(E\{R_{i}\}\right)}^{2} \end{array}$$

By replacing each normal variable *R*_*i*_ by the average of samples from which it is derived and by using $\sum_{i=1}^{n}i=\frac{n(n+1)}{2}$, we have

(3) $$\begin{array}{l} E\{R_{i}\}=\frac{1}{n}(a+(a+\delta )+(a+2\delta )+\ldots +(a+(n-1)\delta )) \end{array}$$

(4) $$\begin{array}{l} \;=\frac{1}{n}(\text{na}+(1+2+\ldots +(n-1))\delta ) \end{array}$$

(5) $$\begin{array}{l} \;=\frac{1}{n}\left(\text{na}+\frac{n(n-1)}{2}\delta \right)=a+\frac{\left(n-1\right)}{2}\delta =\frac{a+b}{2} \end{array}$$

By using $\sum_{i=1}^{n}i^{2}=\frac{n(n+1)(2n+1)}{6}$, we also have

(6) $$\begin{array}{ll} \;E\{R_{i}^{2}\} & =\frac{1}{n}(a^{2}+{\left(a+\delta \right)}^{2}+{\left(a+2\delta \right)}^{2}\\ & \;+\ldots +{\left(a+(n-1)\delta \right)}^{2}) \end{array}$$

(7) $$\begin{array}{l} \;=\frac{1}{n}(na^{2}+2(1+2+\ldots +(n-1))a\delta +(1^{2}+2^{2}\\ \;+\ldots +{\left(n-1\right)}^{2})\delta^{2}) \end{array}$$

(8) $$\begin{array}{l} \;=\frac{1}{n}\left(na^{2}+n(n-1)a\delta +\frac{n(n-1)(2n-1)}{6}\delta^{2}\right) \end{array}$$

(9) $$\begin{array}{l} \;=a^{2}+(n-1)a\delta +\frac{\left(n-1)(2n-1\right)}{6}\delta^{2} \end{array}$$

Using equations 9 and 5, relation 2 leads to

(10) $$\begin{array}{ll} \;\text{Var}\{R_{i}\} & =a^{2}+(n-1)a\delta +\frac{\left(n-1)(2n-1\right)}{6}\delta^{2}\\ & \;-{\left(a+\frac{\left(n-1\right)}{2}\delta \right)}^{2} \end{array}$$

(11) $$\begin{array}{l} \;=(n-1)\left(\frac{2n-1}{6}-\frac{n-1}{4}\right)\delta^{2}=\frac{n^{2}-1}{12}\delta^{2} \end{array}$$

□

**Figure 1:**
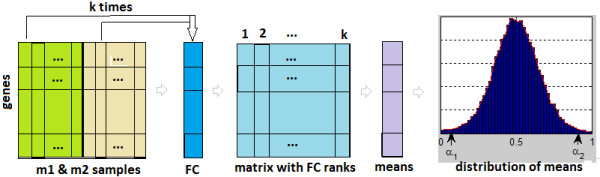
**Steps of the FCROS method.** Scheme depicting the steps of the FCROS method.

Theorem 1 is the basis of our method for selecting the DE genes. The presence of outlier sample(s) will impact the a.o.r. ${\bar{r}}_{i}$ value associated to gene *i*. Thus, we use a trimmed mean by removing a percentage of low and large ranks in the calculation of ${\bar{r}}_{i}$. This percentage is a tuning parameter of the FCROS method which is summarized as follows (see also Figure 1).

#### FCROS algorithm

1. Given microarray data having *m*_1_ control and *m*_2_ test samples, perform *k* ≤ *m*_1_*m*_2_ pairwise comparisons and compute FCs for genes (test/control). These FCs are sorted in increasing order and their corresponding ranks are associated to genes.

2. Compute a robust average of rank ${\bar{r}}_{i}$ for each gene (*i* = 1,2,…,*n*) using its *k* values. This can be done using a trimmed mean. Sort values of $\bar{r}$ by increasing order to get $\bar{r^{s}}$ where ${\bar{r}}_{1}^{s}\leq {\bar{r}}_{2}^{s}\leq \ldots \leq {\bar{r}}_{n}^{s}$.

3. Compute sample mean $\bar{R}=\frac{1}{n}\sum_{i=1}^{n}{\bar{r}}_{i}$ and sample variance ${\hat{\sigma }}_{R}^{2}=\frac{1}{n-1}\sum_{i=1}^{n}{\left({\bar{r}}_{i}-\bar{R}\right)}^{2}$. The minimum average rank is $a={\bar{r}}_{1}^{s}$, and the maximum average rank is $b={\bar{r}}_{n}^{s}$. Compute differences between consecutive terms of ${\bar{r}}_{i}^{s}$ and then derive an estimate for parameter *δ* as the mean of the obtained differences: $\hat{\delta }=\frac{1}{n-1}\sum_{i=1}^{n-1}({\bar{r}}_{i+1}^{s}-{\bar{r}}_{i}^{s})$.

4. Use $\bar{R}$ and ${\hat{\sigma }}_{R}^{2}$ as parameters of a normal distribution and associate probabilities to genes through their ${\bar{r}}_{i}$ values. Since a p-value refers to the probability associated with a hypothesis testing statistic, we call probabilities associated to fold change ranks ordering statistics f-values. A f-value close to 0.5 corresponds to an equally expressed (EE) gene, while down- and up-regulated genes have f-values close to 0 and 1, respectively.

5. Set error levels, *α*_1_ and *α*_2_, for down- and up-regulated genes to select the DE genes.

We use standardized ranks, i.e. each component in **r**_*i*_ is divided by *n*. Hence, the mean and standard deviation in step 3 of the algorihm above should be divided by *n*. In the FCROS algorithm, necessary parameters are computed from the dataset except the trimmed mean percentage parameter noted *trim*. Theorem 1 gives theoretical values for many parameters, more precisely $\delta =\frac{b-a}{n-1}$, $\bar{R}=\frac{b+a}{2n}$ and $\sigma_{R}^{2}=(\frac{1}{12}-\frac{1}{12n^{2}})\delta^{2}$. For the ideal situation (*a* = *δ* = 1, *b* = *n*) theoretical mean and variance are $\frac{1}{2}+\frac{1}{2n}\approx \frac{1}{2}$ and $\frac{1}{12}-\frac{1}{12n^{2}}\approx \frac{1}{12}$, respectively. Let us examine the role of parameters *k*, *δ* and *trim*.

##### 

**Parameter** ***k*** The size of the integer *k* allows to fulfill the conditions to apply the central limit theorem, higher values for *k* being optimal. The maximum value *m*_1_*m*_2_ for *k* is determined by the number of control and test samples in the dataset.

##### 

**Parameter** ***δ*** Parameter *δ* takes its value in the interval [0,1]. The ideal value *δ* = 1 is unlikely to be obtained. A small value of the parameter *δ* leads to a small variance ${\hat{\sigma }}_{R_{i}}^{2}$. This will happen when the difference between upper and lower bounds of the ordered a.o.r ${\bar{r}}_{i}$ becomes smaller, i.e., if the observed changes in the ranks associated with genes are large, so that the a.o.r will tend to move away from the ideal bounds 1 and *n*. We can consider the parameter *δ* as a fraction of the dataset size range: $\delta =\frac{b-a}{n-1}=\frac{n}{n-1}(\beta -\alpha )$ where *β* = *b*/*n* and *α* = *a*/*n*. From this point of view, a value of *δ* equal to 0.98 can correspond to (*b* = 0.99*n*,*a* = 0.01*n*) and is better than a value for *δ* equal to 0.66 which can correspond to the bounds ( $b=\frac{5}{6}n,a=\frac{1}{6}n$) which are more distant from *n* and 1. We provide numerical values for *δ* in Additional file 1: Figures S3 and S5 using synthetic and real microarray datasets.

##### 

**Parameter** ***trim*** To have a robust estimation of the o.a.r ${\bar{r}}_{i}$ we use a fraction of ranks associated to gene *i*. Parameter *trim* allows to delete some ranks from each end (small and high ranks) before computing the mean. Thus, a value for *trim* equal to 0.1 means that 80% of the ranks for gene *i* are used to calculate ${\bar{r}}_{i}$.

## Results and discussion

To evaluate the performance of the FCROS method, we used synthetic and real microarray datasets. We compared the results obtained with our method to those obtained using six other methods: the simple fold change (FC), the weighted average difference (WAD), the rank product (RP), the Student t-test (Ttest), the significant analysis of microarray (SAM) and the t-test relative to a threshold (TREAT) methods. All calculations were performed on the same computer (Personnal Computer equiped with i7-2640M processor, 8GB of RAM, under Microsoft Windows Professional 7) and R version 3.0.1. We implemented a new R package, fcros, which is available from the comprehensive R archive network ( http://cran.r-project.org/web/packages/fcros/) [25]. For all results presented, the *trim* parameter was set to 0.3. We also used three other R packages, samr [26], RankProd [27] and limma [9] with their default settings, but the parameter huge in the RankProd package was set to TRUE. We used the ROC (receiver operating characteristics) R package [28] to obtain an area under a ROC curve (AUC) for methods when true DE genes are available. For real microarray datasets, no prefiltering was performed before searching for the DE genes except for one dataset.

### Synthetic datasets

We used the microarray data simulation model (MADSIM) described in [29] to generate synthetic data with known characteristics; in particular, the indexes of the DE genes are known. A R package implementing MADSIM is available from the comprehensive R archive network [30].

#### 

##### 

**Synthetic datasets 1** To evaluate the behavior of the FCROS method in the presence of noise, we used three different values for the parameter *σ*_*n*_ of MADSIM. 100 simulations corresponding to different initializations (*rseed* = 10, 20, 30, …, 1000.) were used. All other parameters of MADSIM were set to their default settings. More precisely, *m*_1_ = *m*_2_ = 7, *n* = 10,000 and the proportion of DE genes was set to 0.02. These settings lead to an expected number of 200 DE genes. Additional file 1: Figure S1 shows the M-A plot [31] for 3 datasets which correspond to 3 setting values for parameter *σ*_*n*_ of MADSIM.

**Figure 2:**
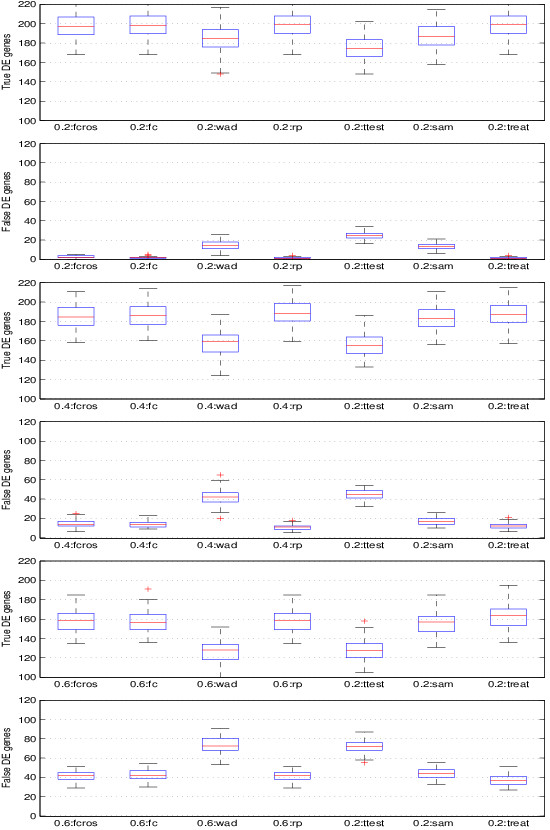
**Comparison of the performance of methods with synthetic data associated with varying levels of noise.** Boxplots of the number of the true and false DE genes using the FCROS (fcros), FC (fc), WAD (wad), RP (rp), Student t-test (ttest), SAM (sam) and TREAT (treat) methods. The noise level parameter *σ*_*n*_ was set to 0.2, 0.4 and 0.6. 100 simulations were used for these results.

For each dataset, i.e. corresponding to a given value for parameters *rseed* and *σ*_*n*_, we used the FCROS and the six other methods to determine the DE genes, the number of which was set to that of true DE genes. The genes selected by each method were split into two sets: true and false DE genes. The results are plotted in Figure 2, which shows that the FCROS, FC, RP, SAM and TREAT methods performed well, and that the Ttest and WAD methods had a lower performance. Of note, in these tests, the runtime of the FCROS method was more than hundred times faster than that of the RP method.

##### 

**Synthetic dataset 2** We used MADSIM to generate a dataset with *m*_1_ = *m*_2_ = 15 and default settings for all other parameters. This dataset has 198 true DE genes. Additional file 1: Figure S2 shows the M-A plot [31] of this synthetic dataset. Synthetic dataset2 was used in different scenarios where we specified different values (*m*_1_*xm*_2_) for the control and test samples, and performed the following steps: a) random selection of *m*_1_ control and *m*_2_ test samples from their respective sets, b) running the FCROS and the six other methods, c) selection of the top 198 DE genes for each method, and assignment of a value of 1 to true DE genes and of 0 to all other genes. These 3 steps are repeated 100 times and the total occurences of 1 for each method and *m*_1_*xm*_2_ combination is calculated as its score, which is thus comprised between 0 and 100.

**Figure 3:**
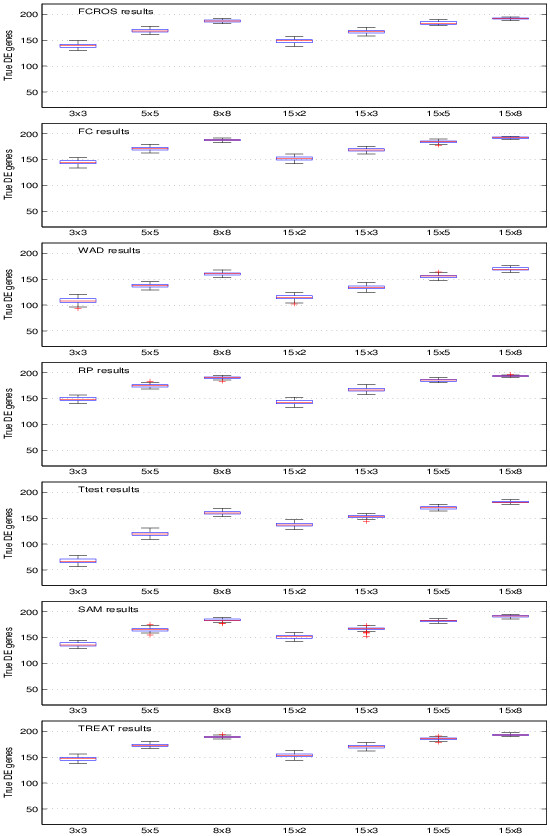
**Comparison of the performance of methods with synthetic data and varying sizes of sample groups.** Number of the true DE genes using the FCROS, FC, WAD, RP, Ttest, SAM and TREAT methods.

Results obtained for the seven methods are shown in Figure 3. The FCROS, FC, RP and TREAT methods had similar power of detection, with the TREAT and RP methods exhibited a slightly better performance for the case 3x3. The Student t-test and the WAD methods gave worse results than the other methods. In addition, we performed another run using the complete dataset (*m*_1_ = 15, *m*_2_ = 15). Table 1 shows the results obtained as well as the AUC (area under a ROC curve) values for the seven methods. The TREAT, FC and RP methods had the lowest error while the Ttest and WAD methods had the highest one.

**Table 1:** Comparative results for the synthetic dataset 2

**Method**	**Thresholds**	**Selection**	**False**	**Error**	**AUC**
FCROS	0.002348226, 0.998	198	2	1.0%	0.9999928
FC	1.5922	198	1	0.5%	0.9999933
WAD	1.2072	198	17	8.5%	0.9981039
RP	0.00009	198	1	0.5%	0.9999985
Ttest	0.00059892	198	6	3%	0.9999356
SAM	0.0007	198	3	1.5%	0.9999691
TREAT	0.00078531	198	1	0.5%	0.9999990

Synthetic dataset 2: Selection, errrors and AUC for the seven methods. Error is calculated as the number of false genes detected divided by 198, the number of true DE genes in the dataset.

We recorded values for parameter *δ* for runs of the FCROS method. Results obtained are plotted in Additional file 1: Figure S3. The values for *δ* are close to 1 if control and test samples are not randomized, see panel A of Additional file 1: Figure S3. These values decrease towards 0.7 for random and an increasing number of control and test samples, see panel B of Additional file 1: Figure S3.

### Microarray data

We used seven microarray datasets to evaluate the performance of the FCROS method. All data were generated with the Affymetrix technology. The first dataset ("Platinum Spike") is from [32] and consists of 18 spike-in samples (9 controls versus 9 tests). This dataset is available from the Gene Expression Omnibus website under the accession number GSE21344. The next six post-processed datasets are available from [33]. For the second dataset, 58 diffuse large B-cell lymphoma (DLBCL) patients and 19 follicular lymphoma patients were used [34]. The third dataset (Prostate) consists of 102 samples using 50 non-tumor and 52 tumor prostate patients [35]. The fourth dataset (Colon) consists of 22 control and 40 colon cancer samples [36]. For the fith dataset (Leukemia), 47 acute lymphoblastic leukemia and 25 acute myeloblastic leukemia patients were used [5]. The sixth dataset (Myeloma) was obtained using 36 patients without and 137 patients with bone lytic lesions [37]. For the seventh dataset (ALL-1), 128 different individuals (95 B-cell leukemia and 33 T-cell leukemia) were used [38].

#### 

##### 

**"Platinum Spike" dataset** We downloaded the Affymetrix CEL format files from the GEO website (GSE21344) and used the RMA (robust multi-array average) method to obtain signals for probes [39]. We downloaded the designated FC associated to probes from: http://www.biomedcentral.com/content/supplementary/1471-2105-11-285-s5.txt (accessed on 23 september 2013). Using this file, we retained 18952 probes, among which 1940 are known as DE. Each of these probes has an observed FC obtained using RMA normalized data and a designated FC read from the file we downloaded. Additional file 1: Figure S4 shows the M-A plot [31] of the "Platinum Spike" dataset.

**Figure 4:**
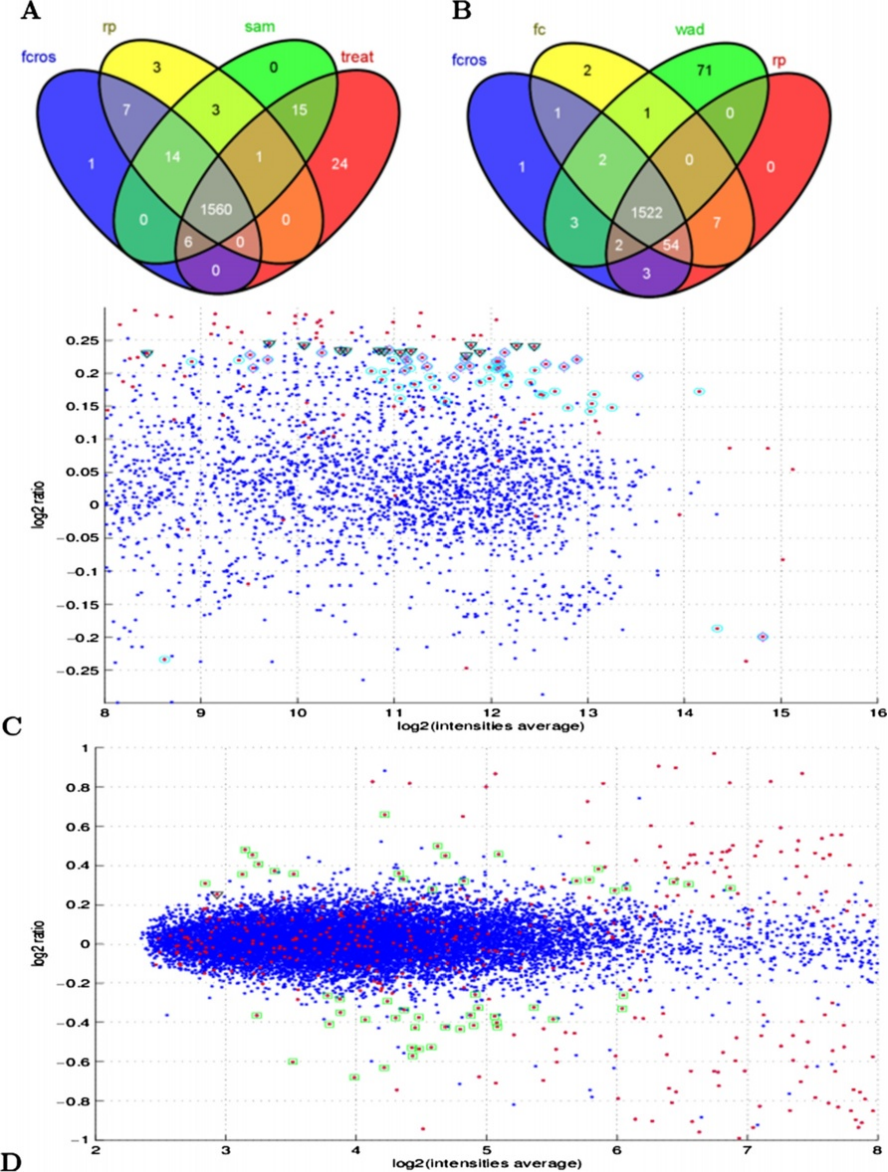
**Comparison of the performance of methods with the Platinum Spike dataset.** Platinum Spike dataset, Venn diagram of top 1940 genes by **(A)** the FCROS, RP, SAM and TREAT methods or **(B)** by the FCROS, FC, WAD and RP methods. Only genes that correspond to true DE genes are represented. M-A plot: **(C)** highly expressed genes where magenta diamonds are used for the 24 genes detected by the TREAT method (panel **A**), black triangles are used for the 15 genes detected by the SAM and TREAT methods (panel **A**), cyan circles are used for the 71 genes detected by the WAD method (panel **B**); and **(D)** weakly expressed genes where green squares are used for the 54 genes detected by the FCROS, FC and RP methods (panel **B**). Additional file 1: Figure S4 shows the full M-A plot of this dataset.

We ran the seven methods and selected the top 1940 probes which were then crossed with the set of the designated DE probes. The results are summarized in Table 2. In this Table, the "Status" indicates whether the gene is equally expressed (EE) or differentially expressed (DE). The "A vs B" is the designated fold change and "Number" is the number of probes for a status. The AUC values and the percentages of false detection for the seven methods are also given. The WAD, Ttest, SAM and TREAT methods were more efficient for A vs B = 0.83 than the other methods (FCROS, FC and RP). For all other A vs B cases, the seven methods have a similar detection efficiency. The results obtained with the FCROS, RP, SAM and TREAT methods are represented in a Venn diagram in Figure 4A. This figure shows that the large majority of DE genes were similarly detected by all 4 methods. The t-test based methods (SAM and TREAT) were slightly more sensitive than the FC-based methods as they detected 39 DE genes (mostly with low FC values, Figure 4C) that neither the FCROS or RP methods detected.

**Table 2:** Comparative results for the Platinum spike dataset

**Status**	**A vs B**	**Number**	**FCROS**	**FC**	**WAD**	**RP**	**Ttest**	**SAM**	**TREAT**
EE	0	13337	216	220	19	221	142	203	176
DE	0.25	192	161	161	158	161	162	161	161
DE	0.28	174	162	163	154	163	157	163	163
DE	0.4	163	132	134	127	133	131	133	133
DE	0.66	189	151	155	147	154	89	149	139
DE	0.83	166	46	43	111	40	118	60	83
EE	1	3426	52	49	232	50	158	56	77
DE	1.5	167	134	134	131	135	114	135	131
DE	1.7	166	150	150	141	150	145	149	149
DE	2	184	161	161	158	162	162	162	161
DE	3	98	94	94	92	94	94	94	94
DE	3.5	445	397	394	382	396	388	393	392
EE/DE	MC	231	74	74	77	73	71	74	73
EE/DE	MF	14	10	8	11	8	9	8	8
True DE genes detected			1588	1589	1601	1588	1560	1599	1606
Error			18.14%	18.09%	17.47%	18.14%	19.58%	17.57%	17.21%
AUC			0.91054	0.90984	0.92030	0.91077	0.90891	0.91084	0.91094

Platinum Spike dataset: number of genes detected by each method for a given change condition. EE and DE are for equally expressed and differentially expressed, respectively. "A vs B" is the designated fold change and "Number" is the number of probes for each change condition. Detection error and the AUC are also given at the bottom of the Table.

We further compared the performances of the 4 FC-based methods, Figure 4B. This comparison revealed that the WAD method outperformed the other methods, as it specifically detected 71 DE genes (Figure 4C). However, it should be noted that the FC, FCROS and RP methods collectively identified 68 DE genes (mostly with low expression values, Figure 4D) that were not detected by the WAD method.

#### 

##### 

**Results for six sets of microarray data** We used the DLBCL, Prostate, Colon, Leukemia, Myeloma and ALL-1 datasets (see above) to compare results obtained with the FCROS method and with the other six methods. To select the list of the most DE genes for each method, we used the results of the RP method, for which a PFP value of zero was associated to some genes. We determined the number of such genes and then set thresholds for the other methods to obtain a similar number of DE genes. We also recorded the runtime of each method. Results are summarized in Table 3. Errors for the Ttest, SAM and TREAT methods are obtained using $100\alpha \frac{n}{n_{2}}$, where *n* is the total number of genes, *α* the threshold used for the selection and *n*_2_ the number of selected DE genes. For the FCROS method the error is given by 100(*α*_1_ + 1 - *α*_2_), where *α*_1_ and *α*_2_ are the selection thresholds.

**Table 3:** Comparative results for six microarray datasets (a)

**Dataset**	**Method**	**Thresholds**	**Selection**	**Runtime (s)**	**Error**
DLBCL	FCROS	0.0228, 0.9773	424	4.79	4.55%
(*n* = 7129)	FC	1.444	425	0.13	na
(*m*_1_ = 58, *m*_2_ = 19)	WAD	1.1575	424	0.14	na
	RP	0	425	656.36	0%
	Ttest	0.0001	428	1.48	0.17%
	SAM	0.00032	427	26.66	0.5%
	TREAT	0.0025	425	0.16	4.19%
Prostate	FCROS	0.0356, 0.9644	1009	19.58	7.12%
(*n* = 12625)	FC	1.27	1008	0.3	na
(*m*_1_ = 50, *m*_2_ = 52)	WAD	1.0805	1010	0.32	na
	RP	0	1010	2491.54	0%
	Ttest	0.00068	1008	2.72	0.85%
	SAM	0.00041	1013	55.6	0.51%
	TREAT	0.0153	1010	0.33	19.12%
Colon	FCROS	0.0187, 0.9802	95	0.99	3.85%
(*n* = 2000)	FC	1.8	95	0.05	na
(*m*_1_ = 22, *m*_2_ = 40)	WAD	1.346	95	0.08	na
	RP	0	96	168.78	0%
	Ttest	0.00015	97	0.5	0.3%
	SAM	0.00055	95	7.08	1.15%
	TREAT	0.00028	95	0.06	0.58%
Leukemia	FCROS	0.028, 0.9717	493	4.7	5.63%
(*n* = 7129)	FC	1.942	494	0.12	na
(*m*_1_ = 47, *m*_2_ = 25)	WAD	1.1668	494	0.17	na
	RP	0	494	768.65	0%
	Ttest	0.00052	494	1.43	0.75%
	SAM	0.00051	494	28.71	0.74%
	TREAT	0.00153	494	0.13	2.2%
Myeloma	FCROS	0.01296, 0.987	450	34.77	2.59%
(*n* = 12625)	FC	1.5189	452	0.4	na
(*m*_1_ = 36, *m*_2_ = 137)	WAD	1.212	449	0.49	na
	RP	0	451	4721.73	0%
	Ttest	0.0051	449	2.9	14.34%
	SAM	0.0055	450	94.84	15.43%
	TREAT	0.0192	451	0.54	53.75%
ALL-1	FCROS	0.0355, 0.9644	1187	22.72	7.11%
(*n* = 12625)	FC	1.4176	1187	0.5	na
(*m*_1_ = 95, *m*_2_ = 33)	WAD	1.1165	1185	0.64	na
	RP	0	1188	3108.43	0%
	Ttest	0.000057	1186	2.66	0.06%
	SAM	0.000145	1187	69.48	0.15%
	TREAT	0.00117	1184	0.26	1.25%

Comparative results for six microarray datasets (na = not available).

The results obtained show that the FC, WAD and TREAT methods have the smallest runtime followed by the Ttest and FCROS methods. The RP method has the largest runtime, which is more than 100 times higher than that of the FCROS method. The selection error (FDR) of each method is also shown in Table 3. Except for the Myeloma dataset, all errors are under 10%. The error value for the RP method is the PFP. For the Prostate, the Myeloma and the ALL-1 datasets, we noted that the RP method detects some genes as down and up regulated at the same time. There are 19, 27 and 6 such genes for the Prostate, Myeloma and ALL-1 datasets, respectively. Most of these genes have a FC close to 1. The bad detection performance of the RP method for these datasets probably comes from the number of permutations (100) used. An increase in this number will lead to an increase of the runtime which is already long. Thus, the RP method is suitable for a small number of samples but is not advisable for larger numbers of samples.

Table 4 shows the numbers of genes detected by all methods (common) and those uniquely detected by each method. The SAM and TREAT methods have the smallest number of genes not detected by any other method. We examined genes detected only by the Ttest, WAD or FC methods. The Ttest method detected some genes with a fold change close to 1. The WAD method detected some highly expressed genes with a small FC but missed other genes with a high FC but low expression values. The FC method detected some genes for which only one sample has a large impact on the average values of all samples. Interestingly, for some datasets (e.g. Myeloma, Prostate, DLBCL), the FCROS method detected a relatively high number of probes not detected by other methods. The proportion of such probes varied highly between datasets, and was as high as 75% of the number of commonly detected probes (Myeloma dataset). This observation suggests that the FCROS method can significantly enrich the number of candidate DE genes.

**Table 4:** Comparative results for six microarray datasets (b)

**Dataset**	**Common**	**FCROS**	**FC**	**WAD**	**RP**	**Ttest**	**SAM**	**TREAT**
DLBCL	149	22	79	54	31	99	5	0
Prostate	308	47	58	81	90	183	1	1
Colon	39	1	9	10	9	10	0	4
Leukemia	191	14	89	102	43	44	11	0
Myeloma	70	53	162	47	124	108	5	1
ALL-1	640	26	86	131	55	131	10	0

Common and specific number of genes detected by the seven methods in the six datasets.

**Figure 5:**
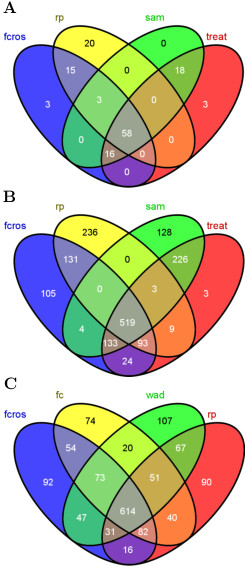
**Comparison of the top seleted genes by different methods for the Colon and the Prostate datasets.** Venn diagram of top seleted DE genes by the FCROS, RP, SAM and TREAT methods: **(A)** Colon and **(B)** Prostate datasets or by **(C)** the FCROS, FC, WAD and RP methods for the Prostate dataset.

We conducted a close inspection of the Colon and the Prostate datasets and used the interactive Venn Diagram plotter software [40] to search for specific and common DE genes detected by the FCROS, RP, SAM and TREAT methods. As expected, the FCROS method shared more genes with the RP method than with the SAM or TREAT methods. Figure 5A shows that 58 genes were detected by these four methods for the Colon dataset. The FCROS method also shared 16 genes with the SAM and the TREAT methods, which were not detected by the RP method. In contrast, no gene was detected jointly by the RP, SAM or TREAT methods which was not detected by the FCROS method. For the Prostate dataset (Figure 5B), 519 genes were detected by all four methods. Many genes were detected only by the FCROS (105) or shared by the FCROS, SAM and TREAT methods (161), and not detected by the RP method. Again, only few genes (12) were detected by the RP and the SAM or TREAT methods that were not detected by FCROS. We further used the Prostate dataset to compare the 4 FC based methods (Figure 5C). We considered that genes that were detected by 2 or more methods are good candidates for true DE genes. There were 1095 such genes, of which 56% were commonly identified by all methods. No method clearly outperformed the other, as each of them failed to detect ≈ 10% of genes that were detected by at least 2 of the other methods. Surpringly, the simple FC method detected the fewest number of genes that were not detected by other methods.

##### 

**Effect of sample number** A literature survey performed in [41] shows that many biological microarray studies use very small number of replicates (e.g. 3 to 5). To evaluate the consequence of such choices on the detection power, we used the Colon dataset, and conducted analyses with varying numbers of control and test samples selected from the original dataset. In a first analysis and for a given (*m*_1_,*m*_2_) pair, we proceeded as follows: a) random selection of *m*_1_ control and *m*_2_ test samples from their true sample groups, b) run the seven methods to obtain results for each, c) select the 100 top DE probes and assign 1 to them and 0 to all other probes. These three steps were repeated 100 times and the total occurences of 1 for each probe was calculated as its score. To set a threshold for the score, we performed a second analysis where control and test samples were chosen without regard for the biological sample groups to which they belong. High scores, in interval [*S*_*thr*_,100], are expected for the DE genes in the first analysis. Small scores, in interval [0,*S*_*thr*_], should be associated to all genes in the second analysis. *S*_*thr*_ is the score threshold which varies with the method used. We sorted genes using their scores in each analysis.

**Figure 6:**
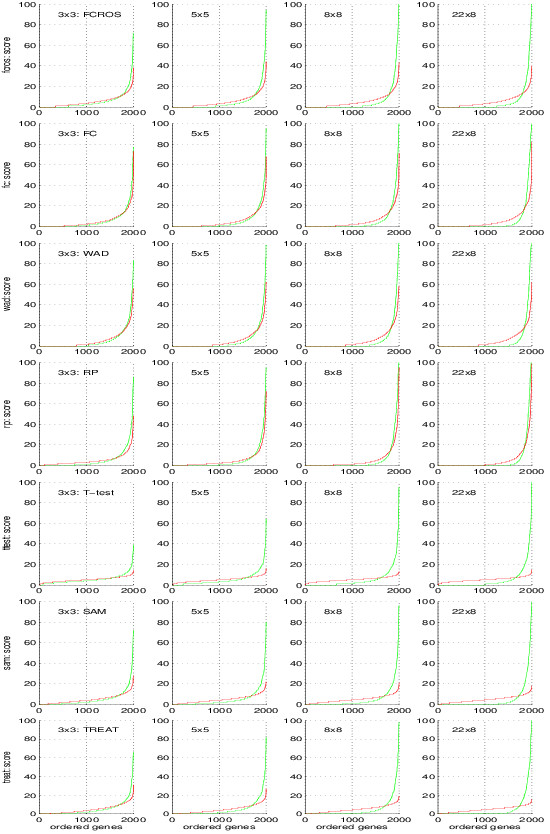
**Impact of sample group size on the performance of the methods.** Colon dataset, plot of scores obtained using the seven methods. Genes in the abscissa are ordered according to their score. The green line is used for the random selection of the control and the test samples from their true sample groups. The red line is used if no distinction was done between true sample groups during the selection of the control and the test samples.

**Figure 7:**
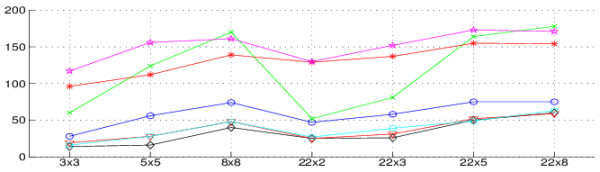
**Number of DE genes selected for the Colon dataset by the seven methods.** Colon dataset, number of DE genes selected using the FCROS (blue circle line), FC (black diamond line), WAD (red triangle line), RP (cyan square line), Student t-test (green x-mark line), SAM (red star line) and TREAT (magenta pentagram line) methods.

Figure 6 shows the results from these analyses. For each setting for control and test samples, we ordered genes according their scores when control and test samples are selected from their true sample groups (green line) or without regard for that true group (red line). Based on these results, we used respectively the score thresholds *S*_*thr*_ of 40, 70, 60, 60, 17, 20 and 18 for the FCROS, FC, WAD, RP, Ttest, SAM and TREAT methods. These thresholds allow to obtain the results illustrated in Figure 7, which shows that rank based methods (FCROS, FC, WAD and RP) select fewer genes than the Ttest, SAM and TREAT methods. The FCROS method detects more genes than the other FC based methods. Results depicted in Figure 6 show that the Ttest, SAM and TREAT methods associated non zero score values to more genes, as revealed by the departure from zero in the x-axis of the score plots. The RP method identifies genes with a high score for completely random control and test samples, indicating its propensity to detect false positives. A similar trend, less pronounced, is also observed for the FC and WAD methods. The WAD method, however, assigned high scores to more genes than the other methods (especially for the 3x3 and 5x5 cases), confirming the high degree of reproducibility of this method [12].

We recorded the values observed for parameters *δ* for all runs of FCROS using different settings for *m*_1_ and *m*_2_. We plotted in Additional file 1: Figure S5 the results obtained for different settings for the number of control and test samples. As for the synthetic datasets, we observed higher values for *δ*, greater than 0.9, when control and test samples are not randomized. However, these values vary more than those obtained with the synthetic datasets. When control and test samples are randomized, values for *δ* decrease towards 0.6 when the number of samples used increases.

##### 

**Analysis of reproducibility** We further used the Colon dataset to assess the reproducibility of DE genes identification in a complex noisy dataset. We conducted 100 runs, in which we used half of the dataset, i.e. 11 control and 20 test samples that were randomly selected. The top 100 genes identified as DE in each run were assigned a score of 1 while all other genes were assigned a score of 0. The overall score for each gene was calculated as the sum of its scores. We evaluated the reproducibility of each method by counting the number of genes with perfect (100) or good (≥90) global scores (Table 5). As expected, the FC based methods were better than the t-test based methods in reproducibly identifying DE genes. Among these methods, FCROS and WAD were more reproducible than FC and RP.

**Table 5:** Comparative results for the Colon dataset

	**Number of genes**						
**Global score**	**FCROS**	**FC**	**WAD**	**RP**	**Ttest**	**SAM**	**TREAT**
100	14	12	13	13	5	5	6
≥90	34	26	37	28	13	17	19

Colon dataset: scores obtained using the seven methods.

## Conclusion

We have described here a new FC-based method and shown that it is powerful to detect DE genes in noisy datasets. Importantly, the FCROS method assigns a statistic to DE genes, which can be used as a selection criterion. FCROS appears to be more specific and much faster than RP, and as sensitive and reproducible as WAD. The FCROS method has two possible applications, when used in combination with other methods: 1) identification of a core set of high confidence DE genes detected by all methods, 2) identification of additional potentially DE genes not detected by other methods. This last possibility may be especially relevant when studying samples with a high degree of intrinsic biological variability (like tumor samples). Our results indeed show that the FCROS method can detect many DE genes in tumor datasets, which escape identification with other methods (Figure 5). In studies of rare diseases, the number of patient samples can be very low while the number of control samples from healthy people is high. The results from Figures 3 and 7 show that the FCROS method performs well in such situations. The FCROS method has also other advantages. (1) It does not require prefiltering to improve the statistic associated with each gene. In contrast, prefiltering is important for other methods, as it decreases the computational load and the FDR. (2) In contrast to the SAM and the RP methods, for which the results can vary from one run to another, the FCROS method is deterministic. (3) The FCROS method can be easily adapted for data originaly from different experiments for which batch related biases can often not be completely corrected by normalization methods. FCROS does not require inter-batch normalization. For instance, if the data are from two experimental batches, we can use *k* = *k*_1_ + *k*_2_ comparisons where *k*_1_ and *k*_2_ are the numbers of pairwise comparisons from the first and the second batch, respectively.

We provide an R package which is deposed on the Comprehensible R Archive Network (CRAN) server for download, see http://www.r-project.org. The function *fcros2()* allows to deal with datasets from two batches. Usage of the package *fcros* is available in the help function.

## Competing interests

The authors declare that they have no competing interests.

## Authors’ contributions

DD drafted the paper and performed the analyses. Both authors developed the method and contributed to the manuscript. Both authors read and approved the final manuscript.

## Supplementary Material

Additional file 1 Supplementary figures. Click here for file
