# Supplementary material for: Fold change rank ordering statistics: a new method for detecting differentially expressed genes
Source: BMC Bioinformatics. 2014 Jan 15;15:14. doi: 10.1186/1471-2105-15-14 (PMC3899927; doi:10.1186/1471-2105-15-14)
Supplement: Additional file 1 — Supplementary figures. [file 1471-2105-15-14-S1.PDF]

Fold change rank ordering statistics: a new method for detecting differentially expressed genes

Doulaye Dembélé[1,2] and Philippe Kastner[1,3]

(1) Institut de Génétique et de Biologie Moléculaire et Cellulaire (IGBMC), INSERM U964, CNRS UMR 7104, Université de Strasbourg, 67404, Illkirch, France

(2) IGBMC Microarray and Sequencing Platform, 67404, Illkirch, France

(3) Faculté de Medécine, Université de Strasbourg, Strasbourg, France

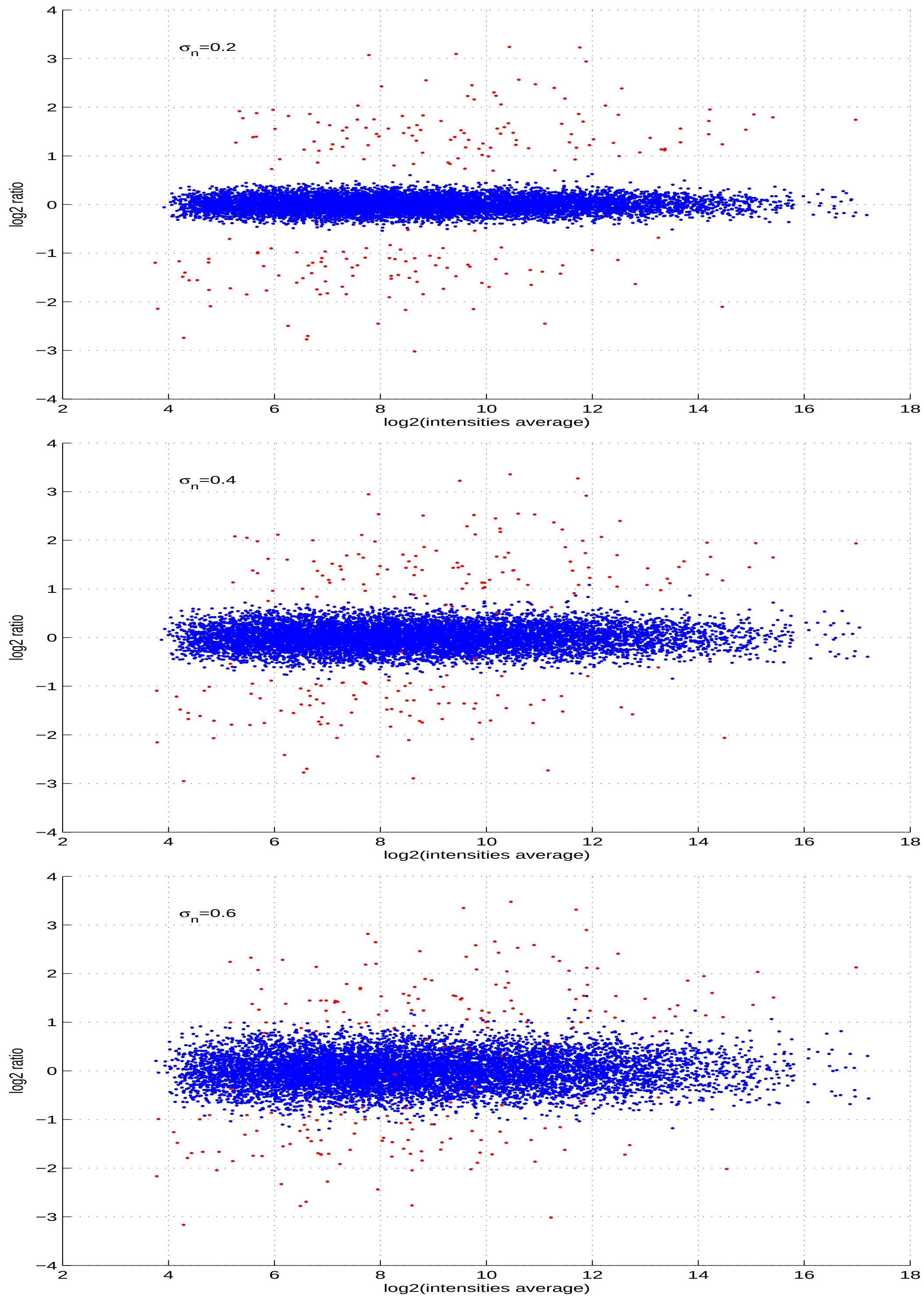

Figure Additional file 1: S1: M-A plot for synthetic datasets 1. These representations are for one run of the R package MADSIM using three noise levels: $\sigma_n = 0.2$, 0.4 and 0.6 (from top to bottom). Parameter rseed in the R package MADSIM was set to 400 and the other parameters are set to their default values. For each plot, we averaged the 7 control and 7 test samples to get only two samples which are used to compute a log_2 ratio (M) and a log_2 intensities average (A) for each gene. Blue dots correspond to equally expressed genes and red dots to differentially expressed genes.

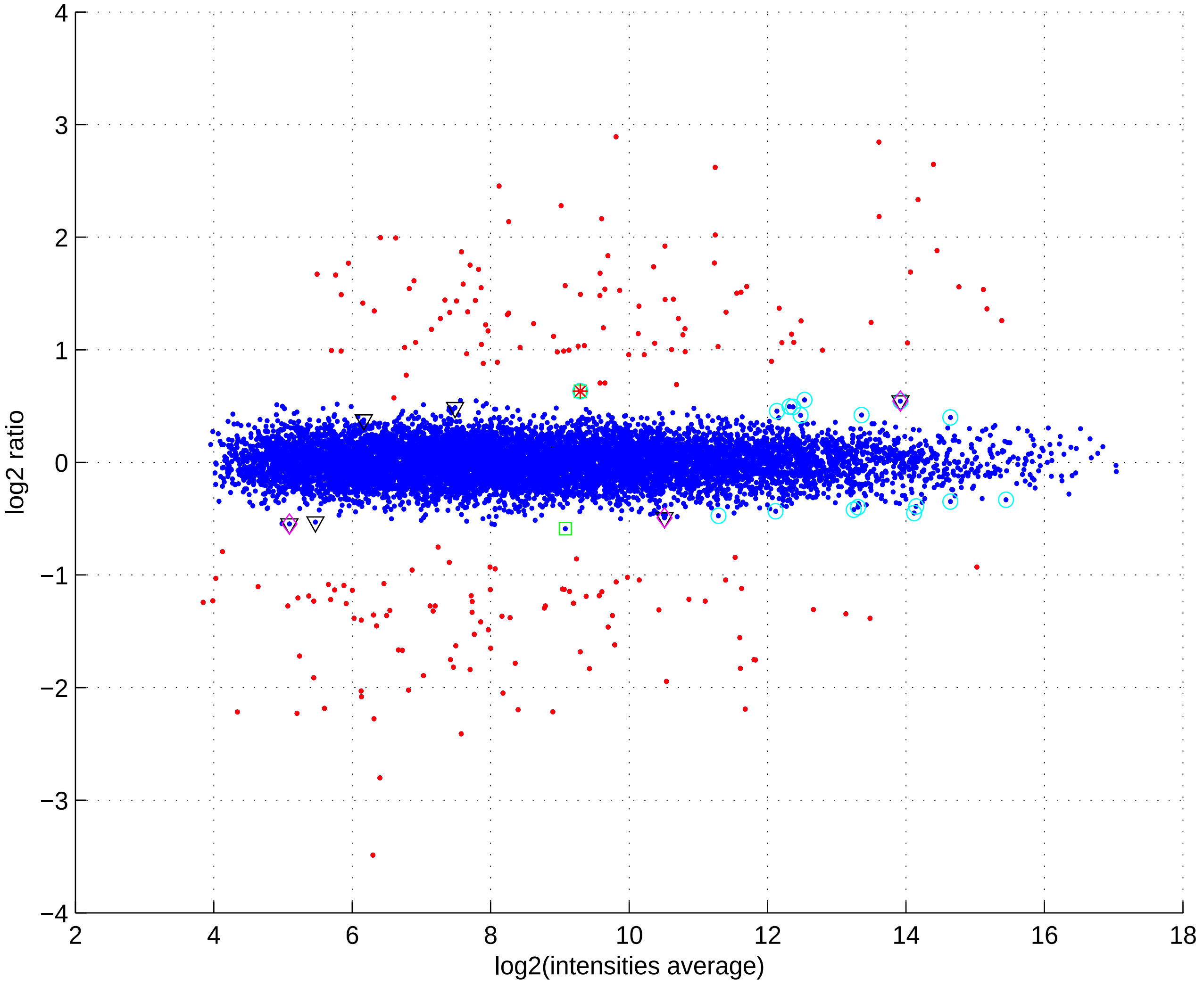

Figure Additional file 1: S2: M-A plot of synthetic dataset2. For this figure, we averaged the 15 control and 15 test samples to get only two samples which are used to compute a log_2 ratio (M) and a log_2 intensities average (A) for each gene. Blue dots correspond to equally expressed genes and red dots to differentially expressed genes. The false detected genes of each method in Table 1 are also shown: green square for FCROS, red star for FC, RP and TREAT (same gene), cyan circle for WAD, black triangle for Ttest and magenta diamond for SAM.

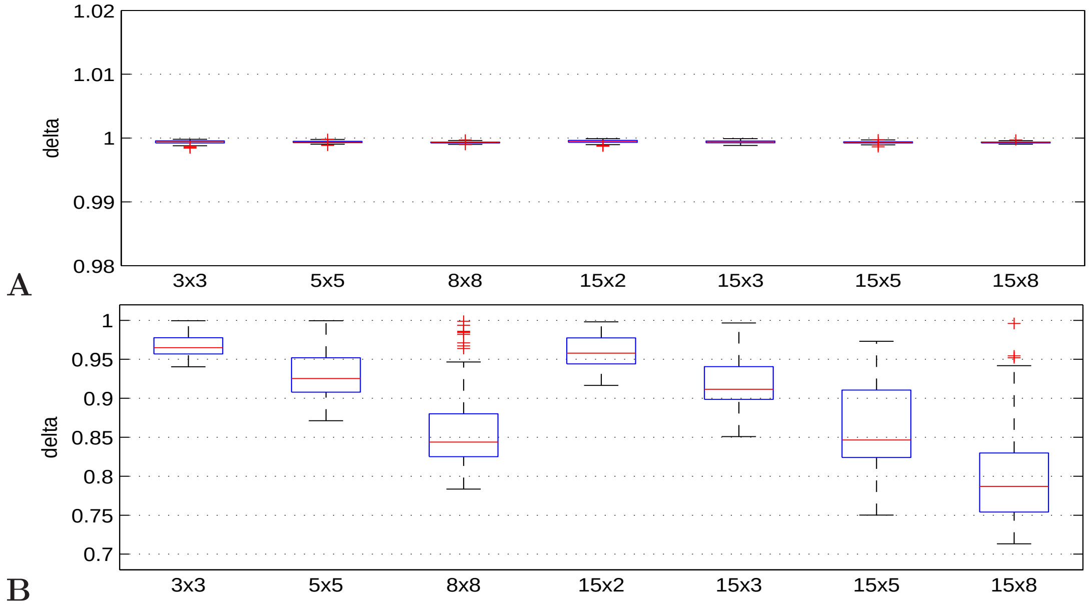

Figure Additional file 1: S3: Boxplots of values obtained for parameters δ when using different settings m_1xm_2 for control and test samples. Control and test samples are randomly selected (A) from their true sample groups or (B) without regard for the biological sample groups.

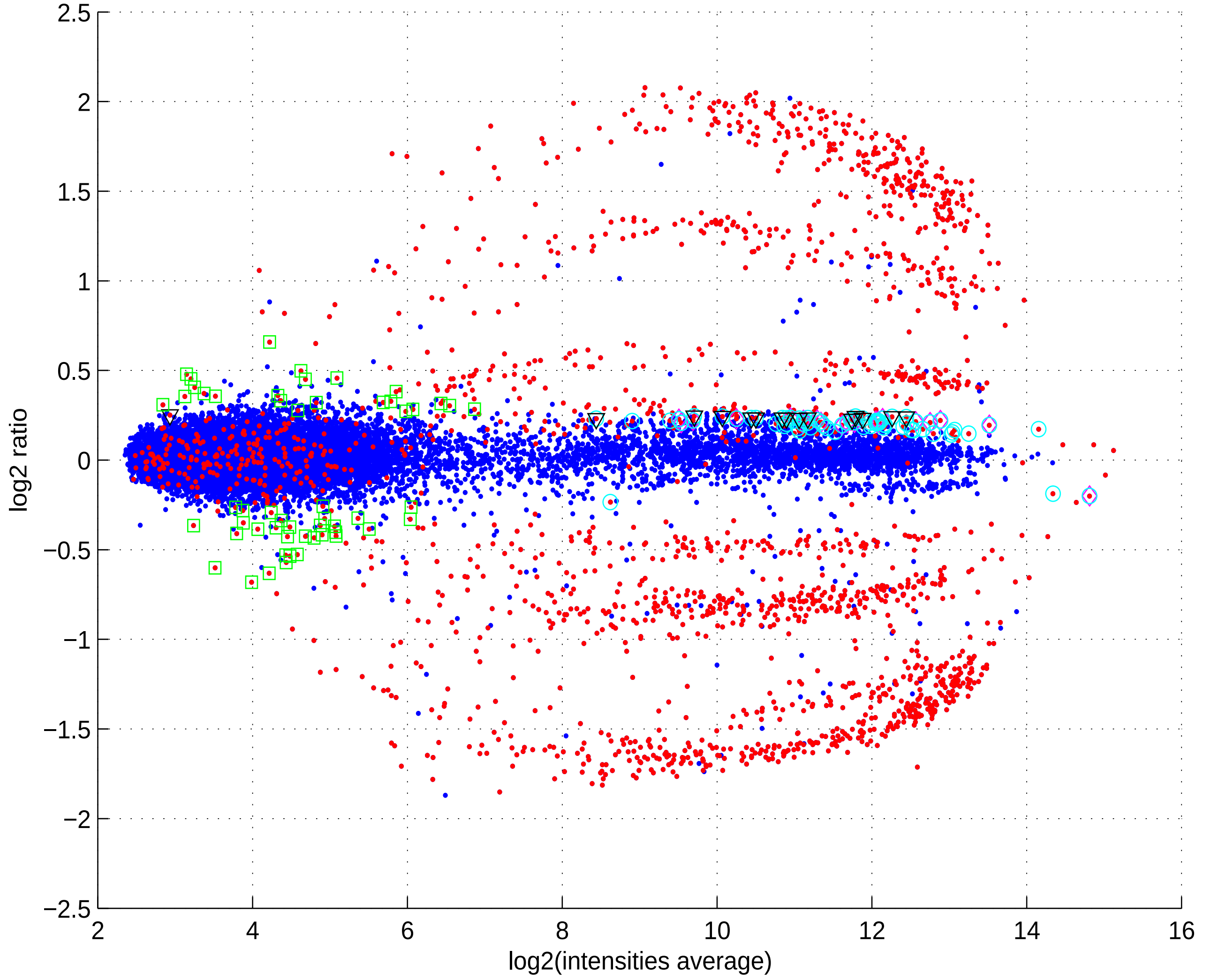

Figure Additional file 1: S4: M-A plot of "Platinum Spike" dataset. For this figure, we averaged the 9 control and 9 test samples to get only two samples which are used to compute a log_2 ratio (M) and a log_2 intensities average (A) for each gene. Blue dots correspond to equally expressed genes and red dots to differentially expressed genes. Some genes from Figure 4 are also represented: magenta diamonds are used for the 24 genes detected by only the TREAT method (Figure 4A), black triangles are used for the 15 genes detected by only the SAM and TREAT methods (Figure 4A), cyan circles are used for the 71 genes detected by only the WAD method (Figure 4B) and green squares are used for the 54 genes detected by only the FCROS, FC and RP methods (Figure 4B).

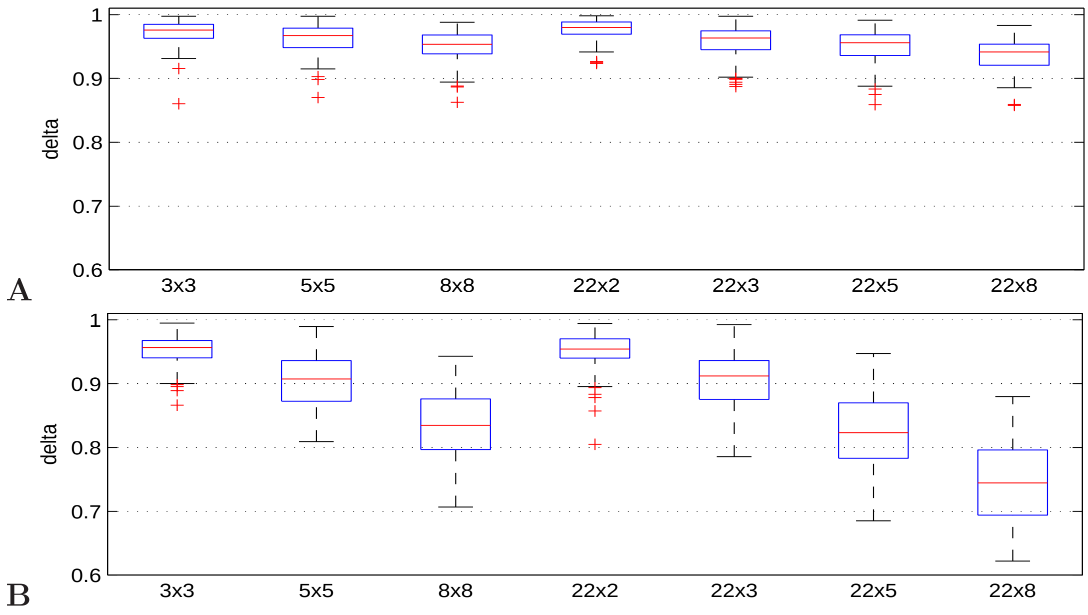

Figure Additional file 1: S5: Boxplots of values obtained for parameters δ when using different settings m_1xm_2 for control and test samples. Control and test samples are selected randomly (A) from their true sample groups (B) without distinction between true sample groups.
